# Editorial: Advances in Microbial Iron Cycling

**DOI:** 10.3389/fmicb.2022.931648

**Published:** 2022-06-21

**Authors:** Lei Yan, Sujun Li, Orit Sivan, Sabine Kasten

**Affiliations:** ^1^Heilongjiang Provincial Key Laboratory of Environmental Microbiology and Recycling of Argo-Waste in Cold Region, College of Life Science and Biotechnology, Heilongjiang Bayi Agricultural University, Daqing, China; ^2^Indiana University Bloomington, Bloomington, IN, United States; ^3^Ben-Gurion University of the Negev Beersheba, Beersheba, Israel; ^4^Alfred Wegener Institute Helmholtz Centre for Polar and Marine Research (AWI) Bremerhaven, Germany; ^5^Faculty of Geosciences, University of Bremen, Bremen, Germany; ^6^MARUM – Center for Marine Environmental Sciences, University of Bremen, Bremen, Germany

**Keywords:** iron cycling, iron redox cycling bacteria, mechanisms, metagenomics, biomineral

Iron is the fourth most abundant element in the Earth's crust and an essential nutrient for almost all living organisms (e.g., Frey and Reed, 2012). As a redox-sensitive transition element, iron is tightly associated with oxidation and reduction processes, and is linked to and also drives the cycling of other elements, including carbon, oxygen, nitrogen, phosphorus, sulfur, and manganese, through numerous processes (Sanyal et al., 2019). These processes are not only actuated by chemical reactions but also driven by microorganisms, which use iron compounds as both electron donors and acceptors and electron transfer in energy generating processes (e.g., Lovley, 1991; Weber et al., 2006; Riedinger et al., 2014; Sivan et al., 2014; Aromokeye et al., 2020).

A large number of microbiological research methods have been developed and shown the essential roles of iron-metabolizing microbes in global biogeochemical cycles (Konhauser et al., 2011). These iron redox cycling bacteria are distributed widely in nature, including marine, forest, and wetland ecosystems (e.g., Hori et al., 2010; Kanaparthi, 2013). Since discovering the first iron-metabolizing bacterium, we have significantly improved our understanding of the diversity, physiology, ecology, and environmental influence of the microorganisms that transform iron. Unraveling the contribution of certain biotic or abiotic processes during iron cycling is highly challenging, despite the availability of various microscopic, spectroscopic, molecular biological and other analytical methods to follow the abiotic and microbial transformation of dissolved, colloidal, and particulate iron redox species (e.g., Raiswell and Canfield, 2012; Kappler et al., 2021).

This Research Topic presents the leading edge of microbial iron cycling, focusing on the geochemical significance of microorganisms and microbial processes which can directly or indirectly contribute to iron cycling in nature. Ten submitted manuscripts had been accepted for publication. In a review article, Lucía et al. described the molecular mechanisms of regulating iron metabolism, focusing on *Saccharomyces cerevisiae*. The budding yeast *S. cerevisiae* has been used as a model organism to study the adaptation of eukaryotic cells to changes in iron availability. Upon iron deficiency, yeast utilizes two transcription factors, Aft1 and Aft2, to activate the expression of a set of genes known as the iron regulon, which are implicated in iron uptake, recycling, and mobilization. At high iron levels, the yeast Yap5, Msn2, and Msn4 transcription factors activate the expression of a vacuolar iron importer called Ccc1, which is the most important high-iron protecting factor devoted to detoxifying excess cytosolic iron that is stored in the vacuole for its mobilization upon scarcity.

Lambrecht et al. described an enrichment culture (BLA1) from meromictic ferruginous Brownie Lake, Minnesota, United States, which contains a Fe(II)-oxidizing Green sulfur bacteria (GSB) and a metabolically flexible putative Fe(III)-reducing anaerobe. “*Candidatus Chlorobium masyuteum*” is closely related to other phototrophic GSB, but distinct in genomic and physiological characteristics. It is able to grow photoautotrophically using Fe(II), and possibly acetate and H^2^. The Fe(III)-reducing anaerobe “*Candidatus Pseudopelobacter ferreus*” grows in anoxic freshwater medium in defined coculture with “*Ca. C. masyuteum*”. Investigation of BLA1 expands the genetic basis for phototrophic Fe(II) oxidation by GSB and highlights the role these organisms may play in Fe(II) oxidation and carbon cycling in ferruginous lakes.

Another article authored by Zhang et al. explored the impact of Fe(III) (Oxyhydr) oxides mineralogy on iron solubilization and associated microbial communities. The authors highlight the influence of Fe mineralogy on the abundance of *Geobacter* and *Shewanella* concerning Fe bio-reduction kinetics in a complex mixture of electron donors.

Garber et al. surveyed the microbial iron cycle in diverse subseafloor habitats using FeGenie (A database and bioinformatics tool that identifies microbial iron cycling genes and enables the development of testable hypotheses on the biogeochemical cycling of iron). They illuminated the diversity of microbial iron redox mechanisms that can occur across various geochemical regimes, suggesting that geochemical constraints likely play an important role in dictating the dominant mechanisms for iron cycling.

Also concerning microbial-mediated iron oxidation and reduction, Yang, Jiang et al. compared biogeographic patterns and composition of the iron redox cycling bacterial community between soil and water samples obtained from different types of peatlands across four regions in Northeast China using high-throughput DNA sequencing. The results suggest that the widespread bacteria involved in iron redox cycling exhibit distinct patterns of distribution and formation mechanisms of microbial community between soil and water in peatlands.

Calapa et al. reported the mass transport of iron and other solutes under flow conditions in subsurface groundwater and its effect on community structure dynamics and Fe(III)-reduction. The results demonstrate that removing inhibitory Fe(II) via mimicking the hydrologic flow of groundwater increases reduction rates and overall Fe-oxide dissolution, which in turn alters the hydrology of the Fe(III)-rich rocks. Reductive weathering of Fe(III)-rich rocks may be more substantial than previously understood.

Yücel et al. studied soluble, colloidal, and particulate iron across the hydrothermal vent mixing zones in Broken Spur and Rainbow, Mid-Atlantic Ridge. The persistence of a nanoparticulate/colloidal phase (retained within 20–200 nm filtrates) even in high-temperature samples, suggested this recalcitrant Fe pool- surviving immediate precipitation-contributes to maintaining high hydrothermal iron fluxes to the deep ocean.

Finally, three articles reported that the iron cycle is closely coupled with carbon, sulfur, nitrogen, phosphorus and manganese cycles. Yang, Li et al. investigated the activity of Anammox bacteria in the Feammox process using the ^15^N isotopic tracing technique combined with 16S rRNA gene amplicon sequencing. A significantly positive relationship between rates of ^15^N_2_ production and Fe(III) reduction indicated the occurrence of Feammox during incubation. They proposed that sole Anammox bacteria or cooperation between Anammox bacteria and Fe(III) reducers serve a potential role in the Feammox process.

Napieralski and Roden investigated the potential interactions among metabolically diverse microorganisms in the near-surface weathering of silicate rocks and confirmed the ability of ferrous iron [Fe(II)] oxidizing bacteria (FeOB) to grow via the oxidation of silicate-bound Fe(II). They suggested that chemolithotrophic and organotrophic microorganisms likely coexist and contribute synergistically to the overall weathering of the *in-situ* bedrock outcrop.

Nabeh et al. investigated and quantified the interactions between iron-sulfide minerals and biomass-derived organic carbon. The amounts of carbon and nitrogen associated with biogenic iron sulfide minerals increased with increasing cell biomass concentrations in the media, indicating that the microbial cells play an essential role in increasing organic carbon concentrations in iron sulfide minerals.

In summary, the collected articles show novel pathways for iron uptake and acquisition, intracellular and extracellular iron mineralization, microbially mediated iron cycling in natural and artificial environments, and advances in the analysis of the effect of the iron cycle on the cycling of other elements. This knowledge broadens our perspective on the iron cycle and will contribute to understanding the transformations of carbon, nitrogen and phosphorus.

## Author Contributions

LY initiated and wrote this Editorial and revised by SL, OS, and SK. All authors contributed to the article and approved the submitted version.

## Conflict of Interest

The authors declare that the research was conducted in the absence of any commercial or financial relationships that could be construed as a potential conflict of interest.

## Publisher's Note

All claims expressed in this article are solely those of the authors and do not necessarily represent those of their affiliated organizations, or those of the publisher, the editors and the reviewers. Any product that may be evaluated in this article, or claim that may be made by its manufacturer, is not guaranteed or endorsed by the publisher.
